# Comparative effectiveness of β-lactam versus vancomycin empiric therapy in patients with methicillin-susceptible *Staphylococcus aureus* (MSSA) bacteremia

**DOI:** 10.1186/s12941-016-0143-3

**Published:** 2016-04-26

**Authors:** Davie Wong, Titus Wong, Marc Romney, Victor Leung

**Affiliations:** PGY-V Infectious Diseases Residency Training Program, Vancouver General Hospital, University of British Columbia, D 452 Heather Pavilion, 2733 Heather Street, Vancouver, BC V5Z 1M9 Canada; Department of Pathology and Laboratory Medicine, University of British Columbia, Vancouver, BC Canada; Medical Microbiology Laboratory, Division of Medical Microbiology and Infection Control, Vancouver General Hospital, JPPN1, 899 W 12th Ave., Vancouver, BC V5Z 1M9 Canada; Medical Microbiology Laboratory, Division of Medical Microbiology, St. Paul’s Hospital, 1081 Burrard St., Vancouver, BC V6Z 1Y6 Canada

**Keywords:** *Staphylococcusaureus*, Bacteremia, Methicillin-susceptible, Vancomycin, Beta-lactam, Empiric therapy

## Abstract

**Background:**

Vancomycin may be inferior to β-lactams for the empiric treatment of methicillin-susceptible *Staphylococcus aureus* (MSSA) bacteremia. We compared empiric β-lactams to vancomycin to assess clinical outcomes in patients with MSSA bacteremia.

**Methods:**

We conducted a retrospective cohort study of adult inpatients with their first episode of MSSA bacteremia at two tertiary care hospitals in Vancouver, Canada, between 2007 and 2014. Exposure was either empiric β-lactam with or without vancomycin or vancomycin monotherapy. All patients received definitive treatment with cloxacillin or cefazolin. The primary outcome was 28-day mortality. Secondary outcomes were 90-day mortality, duration of bacteremia, and hospital length-of-stay. Outcomes were adjusted using multivariable logistic regression.

**Results:**

Of 669 patients identified, 255 met inclusion criteria (β-lactam = 131, vancomycin = 124). Overall 28-day mortality was 7.06 % (n = 18). There were more cases of infective endocarditis in the β-lactam than in the vancomycin group [24 (18.3 %) vs 12 (9.7 %), *p* = 0.05]. Adjusted mortality at 28 days was similar between the two groups (OR 0.85; 95 % CI 0.27–2.67). The duration of bacteremia was longer in the vancomycin group (97.1 vs 70.7 h, *p* = 0.007). Transition to cloxacillin or cefazolin occurred within a median of 68.3 h in the vancomycin group.

**Conclusions:**

Empiric β-lactams was associated with earlier clearance of bacteremia by a median of 1 day compared to vancomycin. Future prospective studies are needed to confirm our findings.

## Background

*Staphylococcus aureus* is the leading cause of bacteremia and carries a mortality of 20–30 % in the twenty-first century [1, 2]. Empiric vancomycin is commonly prescribed when *S. aureus* is isolated from a blood culture but antimicrobial susceptibilities are not yet known, because up to 50–60 % of bloodstream isolates are methicillin-resistant *S. aureus* (MRSA) at some centres [3–8]. However, vancomycin is inferior to semi-synthetic anti-Staphylococcal penicillins (e.g., cloxacillin) and first generation cephalosporins (e.g., cefazolin) for the definitive treatment of methicillin-susceptible *S. aureus* (MSSA) bacteremia [9–11]. Cloxacillin and cefazolin are equally efficacious in treating MSSA bacteremia and are the optimal agents against MSSA [10]. Vancomycin is associated with higher rates of infection-related mortality, re-infection and bacteriologic failure compared to cloxacillin or cefazolin in the definitive treatment of MSSA bacteremia [9, 12–15]. Whether vancomycin is inferior to β-lactams for empiric therapy remains to be fully elucidated. Early studies suggested that empiric vancomycin was associated with worse outcomes compared to empiric β-lactam therapy [3, 16, 17], but more recent data did not demonstrate any differences [15]. Although controversial, some experts recommend the addition of a β-lactam agent to empiric therapy to provide optimal coverage for MSSA in patients at the highest risk of morbidity and mortality from *S. aureus* bacteremia (SAB) [18]. Currently, no studies have compared empiric β-lactam to vancomycin in patients with MSSA bacteremia who are transitioned to cloxacillin or cefazolin for definitive therapy. We assessed if empiric β-lactam with or without vancomycin compared to vancomycin alone was associated with differences in clinical outcomes in patients with MSSA bacteremia who received definitive therapy with cloxacillin or cefazolin.

## Methods

### Patients

We performed a retrospective cohort study of adult inpatients aged 18 and older diagnosed with their first episode of MSSA bacteremia at two tertiary care hospitals in Vancouver, Canada, between January 2007 and December 2014, inclusive. Both hospitals are large academic institutions (955 and 435 beds) affiliated with the University of British Columbia that are served by infectious diseases physicians who share similar treatment strategies for SAB. Consecutive patients were included if they received definitive therapy with either cloxacillin or cefazolin. Patients were excluded if there was missing data for 28-day mortality, no empiric therapy was administered, death occurred within 24 h following diagnosis of bacteremia, or polymicrobial bacteremia. Patients were stratified based on empiric treatment with β-lactams or vancomycin. The β-lactam group received one or more of cloxacillin, cefazolin, β-lactam/β-lactamase inhibitor combination, a third generation cephalosporin or a carbapenem, with or without vancomycin. The vancomycin group was not exposed to any β-lactams until the start of definitive therapy. In both groups, other antimicrobials may have been prescribed during empiric and definitive therapy.

### Definitions

Bacteremia was defined as the isolation of MSSA from one or more blood culture bottles. Bacteremia identified within 48 h of hospital admission was considered community-onset, while bacteremia diagnosed after more than 48 h of hospital admission was deemed hospital-onset. Immunocompromised state was present if any of the following were described: neutropenia (≤1.5 × 10^9^/L), congenital immune deficiencies, or use of immunosuppressants (TNF-α inhibitors, prednisone ≥10 mg/day or its equivalent, cancer chemotherapy, methotrexate, cyclophosphamide, mycophenolate mofetil, calcineurin inhibitors, mTOR inhibitors, azathioprine). Definite infective endocarditis was diagnosed using the modified Duke criteria [19]. The source of bacteremia was either stated explicitly or inferred as the most likely source based on available clinical data and microbiological results. Metastatic complications included infections that occurred distant from the presumed primary source such as septic emboli, mycotic aneurysms, osteoarticular infections, and distant abscesses. Surgical source control included only procedures performed in the operating theatre. Empiric therapy began with the first dose of empiric antibiotics and ended with the start of definitive therapy. Definitive therapy began when antimicrobial susceptibilities were released and one of the following treatments was prescribed: (1) cloxacillin or cefazolin (2) discontinuation of other empiric antibiotics for patients already on cloxacillin or cefazolin empirically, or (3) continuation of empiric cloxacillin or cefazolin. Definitive therapy ended when cloxacillin or cefazolin was stopped. Time to receipt of antibiotics was measured from the time of obtaining the first positive blood culture to the time of the first dose of antibiotic. If a patient was already on antibiotics at the time of the first positive blood culture, the time to receipt of antibiotics was zero.

### Outcomes

Our primary outcome was 28-day all-cause mortality. Secondary outcomes were 90-day all-cause mortality, duration of bacteremia, and hospital length-of-stay (LOS). Time to mortality was measured from the date of the first positive blood culture to the date of death. Duration of bacteremia was the time difference between the first positive blood culture and the first negative blood culture. Patients without follow-up blood cultures were excluded from the analysis for duration of bacteremia. Hospital LOS was measured from the date of the first positive blood culture for MSSA to the date of discharge. Patients who did not survive to hospital discharge were excluded from the hospital LOS analysis.

### Data extraction

Patients with MSSA bacteremia were extracted from the medical microbiology laboratory information systems and medical records were reviewed. A single reviewer collected data on patient demographics and comorbidities, blood culture results and antimicrobial therapy.

### Statistical analysis

Our predicted mortality difference between the β-lactam and vancomycin group was 15 % based on a previous study [17]. We estimated a sample size of 100 for each group to capture a 15 % difference in mortality, assuming a mortality rate of 25 and 10 % in the vancomycin and β-lactam group respectively, with 80 % power at a two-tailed alpha level of 0.05. Baseline categorical variables were described as counts and percentages, and differences between groups were assessed with Chi square or Fisher’s exact tests. Continuous variables were presented as means and standard deviations, or medians and interquartile range. Differences between groups were assessed using parametric t-tests or non-parametric Mann–Whitney U tests, as appropriate. Logistic regression methods were used to model the odds ratio of death in the β-lactam compared to vancomycin group. In order to reduce the small sample size bias, Firth correction method was applied to 28- and 90-day mortality [20]. Linear regression model was conducted for hospital LOS and duration of bacteremia. The two outcomes were log-transformed in the analysis to improve normality of the distribution of residuals. All models were adjusted for age, age-adjusted Charlson-comorbidity index (CCI) [21], infectious diseases consultation, infective endocarditis and time to receipt of empiric antibiotics. The duration of bacteremia was further adjusted for surgical source control. All analyses were performed using the SAS 9.4 software.

### Ethics

The study was approved by the research ethics board at the University of British Columbia, and received institutional approval from Vancouver Coastal Health and Providence Healthcare.

## Results

**Fig. 1:**
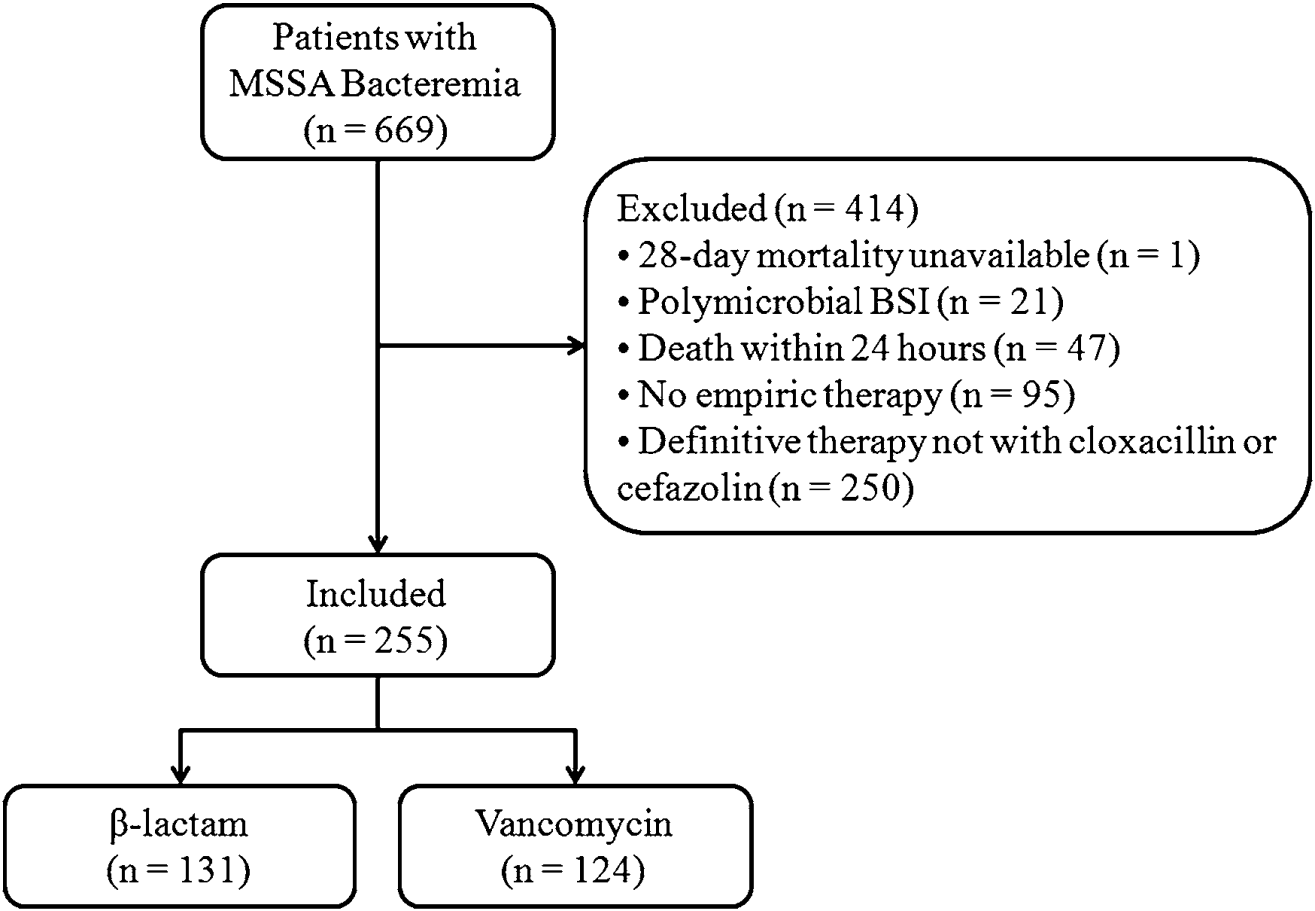
Patient enrollment process. *MSSA* methicillin-susceptible *S. aureus*, *BSI* bloodstream infection

We identified 669 patients with MSSA bacteremia between January 2007 and December 2014, inclusive (Fig. 1). We excluded 414 patients primarily because 60.4 % did not receive cloxacillin or cefazolin for definitive therapy. These patients either remained on broad-spectrum antimicrobials or received vancomycin for definitive therapy due to suspected or confirmed penicillin allergy. Another 22.9 % were not started on empiric therapy. Our cohort consisted of 66.3 % males and 74.1 % of patients had community-onset bacteremia (Table 1). Compared to the β-lactam group, patients in the vancomycin group were older (mean age 59.4 vs 53.2 years, *p* = 0.005), had more medical comorbidities (median CCI 4 vs 3, *p* = 0.001), and were diagnosed with a greater proportion of hospital-onset bacteremia (32.3 vs 19.8 %, *p* = 0.03). Infectious diseases consultation was obtained in most cases (69.8 %), but tended to be higher in the β-lactam (74.8 %) than in the vancomycin group (64.5 %).The most common sources of bacteremia in our cohort were unknown (27.1 %), skin and soft tissue infections (18.4 %), peripheral or central venous catheters (16.5 %) and injection drug use (15.7 %). Infective endocarditis was diagnosed more frequently and surgical source control was achieved more often in the β-lactam (18.3 and 25.2 % respectively) compared to the vancomycin group (9.7 and 15.3 % respectively). The prevalence of infective endocarditis was 14.1 %.

**Table 1:** Comparison of baseline characteristics and clinical outcomes of patients with methicillin-susceptible *S. aureus* bacteremia who received empiric antimicrobial therapy with β-lactams or vancomycin

Patient characteristics	β-lactam^a^ (n = 131)	Vancomycin^a^ (n = 124)	*p* value
Age (mean ± standard deviation in years)	53.2 ± 16.5	59.4 ± 18.3	0.005
Males	83 (63.4)	86 (69.4)	0.31
Community-onset	105 (80.2)	84 (67.8)	0.03
Hospital-onset	26 (19.8)	40 (32.3)	0.03
HIV infection	11 (8.4)	8 (6.5)	0.55
Hepatitis C infection	29 (22.1)	28 (22.6)	0.93
Immunocompromised	14 (10.7)	10 (8.1)	0.47
Alcohol or illicit drug abuse	41 (31.3)	38 (30.6)	0.91
Intravenous drug use	29 (22.1)	29 (23.4)	0.81
Charlson comorbidity index (median with IQR)	3 (1.0–6.0)	4 (1.0–7.0)	0.001
Pitt bacteremia score (median with IQR)	1 (0–2)	1 (0–2)	0.17
Infectious diseases consultation	98 (74.8)	80 (64.5)	0.07
Source of bacteremia			
Central or peripheral line	20 (15.3)	22 (17.7)	0.62
Skin and soft tissue	31 (23.7)	16 (12.9)	0.04
Intravenous drug use	21 (16.0)	19 (15.3)	1.00
Bone or joint infection	18 (13.7)	10 (8.1)	0.16
Lung	4 (3.1)	3 (2.4)	1.00
Other	12 (9.2)	10 (8.1)	0.83
Unknown	25 (19.1)	44 (35.5)	0.005
Infective endocarditis	24 (18.3)	12 (9.7)	0.05
Metastatic complications	40 (30.5)	27 (21.8)	0.11
Surgical source control	33 (25.2)	19 (15.3)	0.05
Recurrent infection at 6 months	4 (3.1)	4 (3.2)	1.00
Empiric antimicrobials			
Vancomycin	88 (67.2)	123 (99.2)	<0.001
Daptomycin	2 (1.5)	0	0.50
Linezolid	2 (1.5)	0	0.50
Cloxacillin or cefazolin	131 (100)	0	<0.001
3rd generation cephalosporin	40 (30.5)	0	<0.001
Piperacillin–tazobactam	33 (25.2)	0	<0.001
Ticarcillin–clavulanic acid	3 (2.3)	0	0.25
Carbapenem	2 (1.5)	0	0.50
Other^b^	47 (35.9)	66 (53.2)	0.01
Blood culture time to positivity (median hours with IQR)	20 (16.5–24.9)	18.3 (15.9–23.5)	0.17
Duration of empiric therapy (median hours with IQR)	54 (42.0–69.0)	48 (29.6–75.8)	0.28
Duration of definitive therapy (median days with IQR)	31.5 (13.0–42.0)	28 (10.0–42.0)	0.17
Time to receipt of empiric therapy (median hours with IQR)	2 (0–7)	20.9 (4.2–28.3)	<0.001
Time to receipt of β-lactam (median hours with IQR)	3 (0.2–16.3)	68.2 (51.5–95.4)	<0.001
Time to receipt of cloxacillin or cefazolin (median hours with IQR)	21.0 (4.4–31.2)	68.3 (51.6–95.4)	<0.001
Primary outcome			
28-day mortality	7 (5.3)	11 (8.9)	0.27
Secondary outcomes			
90-day mortality	14 (10.7)	22 (17.7)	0.11
Duration of bacteremia (median hours with IQR)^c^	70.7 (46.9–119)	97.1 (61.6–148)	0.007
≥3 days	58 (50)	72 (63.2)	0.047
Hospital length of stay (median days with IQR)^d^	17 (11–36)	17 (12–36.5)	0.84

*IQR* interquartile range

^a^Variables are displayed as counts and percentages in parentheses unless otherwise specified

^b^Other antimicrobials used during empiric and definitive therapy included rifampin, aminoglycosides, fluoroquinolones, macrolides, trimethoprim-sulfamethoxazole, and clindamycin

^c^15 and 10 patients from the β-lactam and vancomycin group respectively were excluded from the analysis due to lack of follow-up blood cultures

^d^12 and 19 patients from the β-lactam and vancomycin group respectively were excluded from the analysis due to death during hospital admission

In the β-lactam group, the use of multiple β-lactam antibiotics reflects changes made during empiric therapy, but cloxacillin or cefazolin was continued until the start of definitive therapy. One patient in the vancomycin group received only a partial dose of vancomycin and was counted as not having received it. There was a greater delay in receipt of empiric antimicrobials in the vancomycin group (median 20.9 vs 2 h, *p* < 0.001).Transition to cloxacillin or cefazolin occurred within a median of 68.3 h in the vancomycin group. Among the subgroup of patients who received combination therapy with β-lactam plus vancomycin (88/131), 3rd generation cephalosporins (39.8 %) and piperacillin–tazobactam (34.1 %) were the most common empiric β-lactams prescribed initially (Table 2). Initiation of cloxacillin or cefazolin during the empiric period was delayed in the combination subgroup compared to the subgroup that received β-lactam monotherapy (median 23.1 vs 6.5 h, *p* = 0.001). The combination subgroup had a higher Pitt bacteremia score (median 1 vs 0, *p* = 0.01), received more infectious diseases consultations (80.7 vs 62.8 %, *p* = 0.03), and experienced more metastatic complications (37.5 vs 16.3 %, *p* = 0.02) than the β-lactam monotherapy subgroup. Hospital LOS was shorter in patients who received β-lactam monotherapy (median 14 vs 22 days, *p* = 0.02).

**Table 2:** Comparison of baseline characteristics and clinical outcomes of patients with methicillin-susceptible *S. aureus* bacteremia who received empiric combination therapy with β-lactam plus vancomycin or empiric β-lactam monotherapy

Patient characteristics	β-lactam plus vancomycin^a^ (n = 88)	β-lactam monotherapy^a^ (n = 43)	*p* value
Age (mean ± standard deviation in years)	50.5 ± 16.4	58.9 ± 16.6	0.006
Males	53 (60.2)	30 (69.8)	0.34
Community-onset	74 (84.1)	31 (72.1)	0.16
Hospital-onset	14 (15.9)	12 (27.9)	0.16
HIV infection	9 (10.2)	2 (4.7)	0.34
Hepatitis C infection	19 (21.6)	10 (23.3)	0.83
Immunocompromised	8 (9.1)	6 (14.0)	0.39
Alcohol or illicit drug abuse	29 (33.0)	12 (27.9)	0.69
Intravenous drug use	21 (23.9)	8 (18.6)	0.65
Charlson comorbidity index (median with IQR)	2 (1–4)	3 (1–5)	0.07
Pitt bacteremia score (median with IQR)	1 (0–2)	0 (0–1)	0.01
Infectious diseases consultation	71 (80.7)	27 (62.8)	0.03
Source of bacteremia			
Central or peripheral line	12 (13.6)	8 (18.6)	0.45
Skin and soft tissue	20 (22.7)	11 (25.6)	0.83
Intravenous drug use	16 (18.2)	5 (11.6)	0.45
Bone or joint infection	10 (11.4)	8 (18.6)	0.29
Lung	2 (2.3)	2 (4.7)	0.60
Other	9 (10.2)	3 (7.0)	0.75
Unknown	19 (21.6)	6 (14.0)	0.35
Infective endocarditis	19 (21.6)	5 (11.6)	0.23
Metastatic complications	33 (37.5)	7 (16.3)	0.02
Surgical source control	22 (25)	11 (25.6)	1.00
Recurrent infection at 6 months	2 (2.3)	2 (4.7)	0.60
Empiric antimicrobials			
Daptomycin	0	2 (4.7)	0.11
Linezolid	1 (1.1)	1 (2.3)	0.55
Cloxacillin or cefazolin	88 (100)	43 (100)	1.00
3rd generation cephalosporin	35 (39.8)	5 (11.6)	0.001
Piperacillin–tazobactam	30 (34.1)	3 (7.0)	<0.001
Ticarcillin–clavulanic acid	2 (2.3)	1 (2.3)	1.00
Carbapenem	1 (1.1)	1 (2.3)	0.55
Other^b^	27 (30.7)	20 (46.5)	0.08
Blood culture time to positivity (median hours with IQR)	20 (16.0–24.0)	20.7 (18–27.3)	0.07
Duration of empiric therapy (median hours with IQR)	54.1 (43.0–71.7)	51.7 (38–64.5)	0.18
Duration of definitive therapy (median days with IQR)	38 (16–43)	23 (12–40)	0.08
Time to receipt of empiric therapy (median hours with IQR)	1.91 (0–6.17)	3.42 (0.58–17.2)	0.19
Time to receipt of β-lactam (median hours with IQR)	2.88 (0.21–15.1)	3.42 (0.58–17.2)	0.87
Time to receipt of cloxacillin or cefazolin (median hours with IQR)	23.1 (13.0–31.7)	6.5 (1.5–22.1)	0.001
Primary outcome			
28-day mortality	5 (5.7)	2 (4.7)	1.00
Secondary outcomes			
90-day mortality	10 (11.4)	4 (9.3)	1.00
Duration of bacteremia (median hours with IQR)^c^	71.4 (50.3–126.9)	68.5 (36.4–115.8)	0.35
≥3 days	41 (46.6)	17 (39.5)	0.46
Hospital length of stay (median days with IQR)^d^	22 (12–44)	14 (10–22.8)	0.02

*IQR* interquartile range

^a^Variables are displayed as counts and percentages in parentheses unless otherwise specified

^b^Other antimicrobials used during empiric and definitive therapy included rifampin, aminoglycosides, fluoroquinolones, macrolides, trimethoprim-sulfamethoxazole, and clindamycin

^c^6 and 9 patients from the β-lactam plus vancomycin and β-lactam monotherapy subgroup respectively were excluded from the analysis due to lack of follow-up blood cultures

^d^9 and 3 patients from the β-lactam plus vancomycin and β-lactam monotherapy subgroup respectively were excluded from the analysis due to death during hospital admission

The adjusted odds ratio of death at 28 and 90 days between the β-lactam and vancomycin group was 0.85 (95 % CI 0.27–2.67) and 0.88 (0.36–2.17) respectively (Table 3). The overall 28- and 90-day mortality was 18 (7.06 %) and 36 (14.1 %) respectively. Among patients with infective endocarditis, 28- and 90-day mortality was 2 (8.33 %) and 3 (12.5 %) in the β-lactam group and 0 and 1 (8.33 %) in the vancomycin group. No mortality differences were observed between the two hospitals. The duration of bacteremia was shorter in the β-lactam than in the vancomycin group (median 70.7 vs 97.1 h, *p* = 0.007) with an adjusted ratio of mean of 0.77 (95 % CI 0.62–0.95). Hospital LOS was similar between the two groups with an adjusted ratio of mean of 0.86 (95 % CI 0.66–1.10).

**Table 3:** Outcome analysis comparing empiric β-lactam versus vancomycin, adjusted for age, age-adjusted Charlson-comorbidity index, infectious diseases consultation, infective endocarditis and time to receipt of empiric antibiotics

Outcomes	Crude OR (95 % CI)	*p* value	Adjusted OR (95 % CI)	*p* value
28-day mortality	0.60 (0.23–1.55)	0.29	0.85 (0.27–2.67)	0.78
90-day mortality	0.56 (0.28–1.15)	0.11	0.88 (0.36–2.17)	0.79

	Ratio of mean (95 % CI)	*p* value	Adjusted ratio of mean (95 % CI)	*p* value
Duration of bacteremia	0.77 (0.64–0.93)	0.01	0.77 (0.62–0.95)	0.01
Hospital length-of-stay	0.85 (0.68–1.07)	0.16	0.86 (0.66–1.10)	0.23

Duration of bacteremia was further adjusted for surgical source control

*OR* odds ratio, *CI* confidence interval

## Discussion

The goal of our study was to assess if empiric β-lactams with or without vancomycin compared to vancomycin alone was associated with differences in outcomes in patients with MSSA bacteremia. We found no differences in all-cause mortality at 28 and 90 days, or hospital LOS between these two groups. Clearance of bacteremia was delayed by a median of 1 day in the vancomycin group. However, this outcome may have been confounded by the earlier receipt of empiric antibiotics in the β-lactam group (median 2 h). When we analyzed a subset of patients from the vancomycin group (n = 43) whose median time to receipt of empiric antibiotics was 1.97 h, the duration of bacteremia was still longer compared to the β-lactam group, but just shy of statistical significance (95.1 vs 70.7 h, *p* = 0.06), likely because of the reduced sample size. Therefore, it does not appear that time to receipt of empiric therapy had a major impact on time to clearance of bacteremia in our study. Despite the high prevalence of MRSA at both of our institutions (25 and 38 %), only 67.2 % of patients in the β-lactam group received vancomycin empirically as well. Perhaps the awareness of MRSA was low among some treating clinicians or patients who did not receive empiric vancomycin were judged to be at low risk for MRSA infection.

Interestingly, the differential time delay in receipt of empiric antimicrobials was unexpected. Patients in the β-lactam group generally received antibiotics well before the blood culture became positive, while patients in the vancomycin group tended to receive antibiotics shortly after the blood culture turned positive. The reason for this observation is likely multifactorial. First, β-lactam patients generally had more identifiable sources of bacteremia (i.e. more skin and soft tissue infections). Second, the higher prevalence of community-onset bacteremia suggests these patients may have had their first medical contact with the emergency department where sepsis protocols facilitated timely administration of antibiotics.

In the β-lactam group, patients who received empiric β-lactam plus vancomycin had a higher Pitt bacteremia score, experienced more metastatic complications and stayed in hospital longer than those who received empiric β-lactam monotherapy. The greater severity of illness in this combination subgroup may explain the initial use of broad-spectrum β-lactams (ceftriaxone or piperacillin–tazobactam), with subsequent de-escalation to cloxacillin or cefazolin during the empiric period by the infectious diseases consultant when *S. aureus* was identified in the blood culture. De-escalation occurred within a median of 23.1 h, which follows the time to positivity of the first blood culture (median 20 h). Despite differences in baseline characteristics and antimicrobials prescribed, mortality rates and time to clearance of bacteremia were similar between these subgroups.

Our study outcomes were similar to those reported in the literature. The overall 28- and 90-day mortality in our study was low at 18 (7.06 %) and 36 (14.1 %) respectively, but is within the range of 3.6–51.7 % described in a meta-analysis of patients with MSSA bacteremia from catheter-related infections and infective endocarditis by Cosgrove et al. [22]. Definite infective endocarditis was diagnosed in 36 (14.1 %) of our patients, which is similar to rates reported in previous studies [10, 14, 16, 23, 24].

The median duration of bacteremia was longer in the vancomycin compared to the β-lactam group (4 vs 3 days) in our study. In a similar study by Khatib et al. [3], clearance of bacteremia was delayed (duration ≥3 days) more often in patients who received empiric vancomycin (57.6 %) compared to those who received empiric β-lactams (37.5 %). They reported no difference in all-cause or attributable mortality between groups.

We did not find any differences in mortality between treatment groups in our study. In contrast, Lodise et al. [17] demonstrated that empiric β-lactam was associated with lower infection-related mortality than with empiric vancomycin monotherapy (11.4 vs 39.3 %, *p* = 0.005) among injection drug users with predominantly right-sided MSSA infective endocarditis. Even when patients were switched from vancomycin to a semi-synthetic penicillin within a median of 3 days, infection-related mortality remained high at 40.9 %. The overall mortality in Lodise’s cohort was unusually high at 22.2 % compared to a rate of 0–4 % described in a systematic review by Yung et al. [25]. The largest study to date by McDanel et al. revealed that empiric β-lactam therapy (predominantly piperacillin–tazobactam and ceftriaxone) compared to vancomycin was not associated with differences in mortality in patients with MSSA bacteremia [15]. However, this study excluded patients who received empiric vancomycin plus β-lactams, did not address microbiological cure, and evaluated empiric regimens independent of the definitive antimicrobial therapy prescribed.

We included a large proportion of patients who received empiric treatment with optimal anti-MSSA agents (cloxacillin or cefazolin), whereas previous observational studies have either failed to specify the β-lactam agents used or enrolled patients who received mostly broad-spectrum β-lactams. This is an important point because not all β-lactams have equal efficacy against MSSA. As demonstrated in one study, empiric cefazolin or cloxacillin was associated with improved short-term survival compared to empiric regimens containing other β-lactams [26]. Therefore, the ideal study is one that compares a semi-synthetic anti-Staphylococcal penicillin or cefazolin to vancomycin.

Our study has several limitations. The reason for the lack of difference in the primary outcome is likely multifactorial. Because of the low event rate in both groups, our study was potentially underpowered to detect a significant difference in mortality. The lower than expected death rate may be partly due to the exclusion of patients who died within 24 h of the diagnosis of SAB and of patients who remained on broad-spectrum β-lactams. This group may have represented a sicker population and thus, we may have selected for less critically ill patients. The absence of matching with respect to baseline characteristics and the retrospective nature of the study may have also contributed to a lack of difference in the primary outcome. Obtaining subsequent blood cultures was often delayed, which may have led to an overestimation of the duration of bacteremia. However, this effect was likely balanced between both groups. Data regarding adverse effects were not collected due to the difficulty of establishing drug-related events in a retrospective study. A randomized controlled trial would be ideal to address our study question because it would provide better matching of patient baseline characteristics and control of antimicrobials prescribed, and permit prospective monitoring of adverse drug effects. In such a study, daily blood cultures would need to be collected to determine the exact date of clearance of bacteremia. As well, more accurate estimation of the expected mortality rates between groups would be needed when calculating the required sample size. Collaboration between the medical microbiology laboratory, infectious diseases service and antimicrobial stewardship team is essential to execute such a trial.

Until we have more concrete evidence from future prospective studies, the benefit of adding a β-lactam to empiric therapy for MSSA bacteremia remains unclear. Ultimately, the choice of empiric regimen will depend on patient factors, the prevalence of MRSA in the population, and the ability of the microbiology laboratory to rapidly differentiate MSSA from MRSA.

In conclusion, empiric therapy with β-lactams was associated with earlier clearance of bacteremia by a median of 1 day compared to vancomycin, but was not associated with differences in all-cause mortality or hospital LOS in patients with MSSA bacteremia. Our data should be interpreted with caution however, as major differences in the baseline characteristics between the groups may have overshadowed any potential treatment effect. Future prospective studies are needed to confirm our findings. For now, empiric treatment with vancomycin is reasonable if the prevalence of MRSA is significant. The addition of a β-lactam agent could be considered in critically ill patients.
