# Editorial Expression of Concern: Comparative effectiveness of β-lactam versus vancomycin empiric therapy in patients with methicillin-susceptible *Staphylococcusaureus* (MSSA) bacteremia

**DOI:** 10.1186/s12941-020-00394-8

**Published:** 2020-11-17

**Authors:** Davie Wong, Titus Wong, Marc Romney, Victor Leung

**Affiliations:** 1 PGY-V Infectious Diseases Residency Training Program, Vancouver General Hospital, University of British Columbia, D 452 Heather Pavilion, 2733 Heather Street, Vancouver, BC V5Z 1M9 Canada; 2 Department of Pathology and Laboratory Medicine, University of British Columbia, Vancouver, BC Canada; 3 Medical Microbiology Laboratory, Division of Medical Microbiology and Infection Control, Vancouver General Hospital, JPPN1, 899 W 12th Ave., Vancouver, BC V5Z 1M9 Canada; 4 Medical Microbiology Laboratory, Division of Medical Microbiology, St. Paul’s Hospital, 1081 Burrard St., Vancouver, BC V6Z 1Y6 Canada

## Expression of Concern: Ann Clin Microbiol Antimicrob (2016) 15:27 10.1186/s12941-016-0143-3

The Editors in Chief are issuing this Expression of Concern to alert readers to a number of issues with respect to this article [1].

After publication of this article [1], it has come to the attention of the Editors that there is significant text overlap with [2], although both articles present different conclusions. A subsequent investigation has found that article [2] presents research on an increased group of patients performed solely by the first author, Davie Wong and published in parallel with [1]. Authorship of [2] has now been corrected.^1^

Article [1] is a comparison of cloxacillin/cefazolin vs vancomycin in the empiric treatment of MSSA bacteremia, whereas article [2] presents a comparison of beta-lactams (including cloxacillin/cefazolin, ceftriaxone, piptazo, etc.) vs vancomycin. The difference in the conclusions with article [2] may be due to the additional patient data added to study [2]. The institution where this research was carried out has been unable to confirm this. Therefore the Editor advises that readers interpret the data presented with caution.

To further clarify, in article [1] the "Methods" section under "Patients" subsection, the line that reads "The β-lactam group received one or more of cloxacillin, cefazolin, β-lactam/β-lactamase inhibitor combination, a third generation cephalosporin or a carbapenem, with or without vancomycin" should read "The β-lactam group received either empiric cloxacillin or cefazolin, with or without vancomycin" to indicate that receipt of cloxacillin or cefazolin is a strict inclusion criterion.

Davie Wong, Titus Wong, and Victor Leung agree to this Editorial Expression of concern. Marc Romney did not respond to the correspondence regarding this Editorial Expression of concern.

## Footnotes

1 Erratum to: Wong D. Comparison of outcomes in patients with methicillin-susceptible *Staphylococcus aureus* (MSSA) bacteremia who are treated with β-lactam vs vancomycin empiric therapy: a retrospective cohort study. BMC Infect Dis.
